# Crystal structures of three co-crystals of 1,2-bis­(pyridin-4-yl)ethane with 4-alk­oxy­benzoic acids: 4-eth­oxy­benzoic acid–1,2-bis­(pyridin-4-yl)ethane (2/1), 4-*n*-propoxybenzoic acid–1,2-bis(pyridin-4-yl)ethane (2/1) and 4-*n*-but­oxy­benzoic acid–1,2-bis­(pyridin-4-yl)ethane (2/1)

**DOI:** 10.1107/S2056989015019349

**Published:** 2015-10-17

**Authors:** Yohei Tabuchi, Kazuma Gotoh, Hiroyuki Ishida

**Affiliations:** a Department of Chemistry, Faculty of Science, Okayama University, Okayama 700-8530, Japan

**Keywords:** crystal structure, 1,2-bis­(pyridin-4-yl)ethane, 4-alk­oxy­benzoic acid, hydrogen-bonded liquid crystal

## Abstract

The crystal structures of three hydrogen-bonded co-crystals of 4-alk­oxy­benzoic acid–1,2-bis­(pyridin-4-yl)ethane (2/1), namely, 2C_9_H_10_O_3_·C_12_H_12_N_2_, (I), 2C_10_H_12_O_3_·C_12_H_12_N_2_, (II), and 2C_11_H_14_O_3_·C_12_H_12_N_2_, (III), have been determined at 93, 290 and 93 K, respectively. In (I), the asymmetric unit consists of one 4-eth­oxy­benzoic acid mol­ecule and one half-mol­ecule of 1,2-bis­(pyridin-4-yl)ethane, which lies on an inversion centre. In (II) and (III), the asymmetric units each comprise two crystallographically independent 4-alk­oxy­benzoic acid mol­ecules and one 1,2-bis­(pyridin-4-yl)ethane mol­ecule. In each crystal, the two components are linked by O—H⋯N hydrogen bonds, forming a linear hydrogen-bonded 2:1unit of the acid and the base. Similar to the structure of 2:1 unit of (I), the units of (II) and (III) adopt nearly pseudo-inversion symmetry. The 2:1 units of (I), (II) and (III) are linked *via* C—H⋯O hydrogen bonds, forming tape structures.

## Abstract 2

Crystal structures of three co-crystals of 1,2-bis­(pyridin-4-yl)ethane with 4-alk­oxy­benzoic acids have been determined. The asymmetric unit of each compound comprises two crystallographically independent acid mol­ecules and one base mol­ecule, which are held together by O—H⋯N hydrogen bonds, forming linear hydrogen-bonded 2:1 units.

## Chemical context   

Co-crystals of 4-alk­oxy­benzoic acid–4,4′-bipyridyl (2/1), in which the two acids and the base are held together by inter­molecular O—H⋯N hydrogen bonds, show thermotropic liquid crystallinity (Kato *et al.*, 1990 ▸, 1993 ▸; Grunert *et al.*, 1997 ▸). Recently, we have reported the crystal structures of the three compounds of 4-eth­oxy-, 4-*n*-prop­oxy- and 4-*n*-but­oxy­benzoic acid (Tabuchi *et al.*, 2015 ▸). As an expansion of our work on the structural characterization of hydrogen-bonded co-crystals which exhibit liquid phases, we have prepared compounds of 4-alk­oxy­benzoic acid–1,2-bis­(pyridin-4-yl)ethane (2/1) and analyzed the crystal structures. DSC (differential scanning calorimetry) and polarizing microscope measurements show that the compounds of 4-meth­oxy-, 4-eth­oxy- and 4-*n*-propoxybenzoic acid have nematic phases at 419 (1), 421 (1) and 419 (1) K, respectively, while the compound of 4-*n*-but­oxy­benzoic acid exhibits a smectic A phase at 413 (1) K and a nematic phase at 419 (1) K.
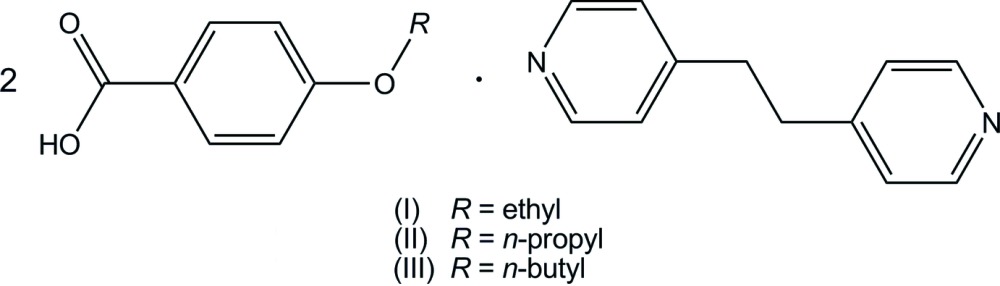


We present here three structures of 4-eth­oxy­benzoic acid–1,2-bis­(pyridin-4-yl)ethane (2/1), (I), 4-*n*-propoxybenzoic acid–1,2-bis­(pyridin-4-yl)ethane (2/1), (II), and 4-*n*-but­oxy­benzoic acid–1,2-bis­(pyridin-4-yl)ethane (2/1), (III). The structure of 4-meth­oxy­benzoic acid–1,2-bis­(pyridin-4-yl)ethane (2/1) has been reported recently (Mukherjee & Desiraju, 2014 ▸).

## Structural commentary   

**Figure 1:**
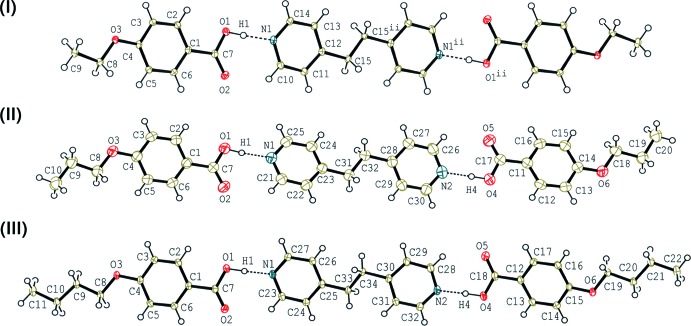
The mol­ecular structures of compounds (I), (II) and (III) determined at 93, 290 and 93 K, respectively, with the atom labelling. Displacement ellipsoids are drawn at the 50% probability level. O—H⋯N hydrogen bonds are indicated by dashed lines [symmetry code for (I): (ii) −*x*, −*y* + 2, −*z*].

The mol­ecular structures of (I), (II) and (III) are shown in Fig. 1 ▸. The asymmetric unit of (I) consists of one 4-eth­oxy­benzoic acid mol­ecule and one half-mol­ecule of 1,2-bis(pyridin-4-yl)ethane which lies on an inversion centre. The two acid mol­ecules and the base mol­ecule are held together *via* O—H⋯N hydrogen bonds (Table 1 ▸) to afford a centrosymmetric linear 2:1 unit. The hydrogen-bonded asymmetric unit is approximately planar with dihedral angles of 9.40 (11), 4.38 (11) and 2.76 (9)°, respectively, between the N1/C10–C14 and O1/C7/O2 planes, the O1/C7/O2 and C1–C6 planes, and the C1–C6 and O3/C8/C9 planes.

The asymmetric units of (II) and (III) are each composed of two crystallographically independent 4-alk­oxybenzoic acid mol­ecules and one 1,2-bis­(pyridin-4-yl)ethane mol­ecule, and the two acids and the base are held together by O—H⋯N hydrogen bonds (Tables 2 ▸ and 3 ▸), forming a linear hydrogen-bonded 2:1 aggregate. Similar to the 2:1 unit of (I), the units of (II) and (III) adopt nearly pseudo-inversion symmetry. The dihedral angles between the pyridine rings of 1,2-bis­(pyridin-4-yl)ethane are 14.36 (6) and 29.92 (7)°, respectively, for (II) and (III). The pyridine ring and the carboxyl group hydrogen-bonded to it are twisted with respect to each other. In (II), the dihedral angles between the N1/C21–C25 and O1/C7/O2 planes, and the N2/C26–C30 and O4/C17/O5 planes are 4.86 (14) and 7.71 (14)°, respectively, while those in (III) are 9.48 (16) and 25.25 (17)°, respectively, between the N1/C23–C27 and O1/C7/O2 planes, and the N2/C28–C32 and O4/C17/O5 planes.

The mol­ecular structures of 4-*n*-prop­oxy- and 4-*n*-but­oxy­benzoic acids in (II) and (III) are approximately planar. The dihedral angles made by the benzene ring with the carboxyl group and the alk­oxy group in each propoxybenzoic acid in (II) are 9.20 (14), 4.36 (14), 1.80 (11) and 5.98 (11)°, respectively, between the C1–C6 and O1/C7/O2 planes, the C11–C16 and O4/C17/O5 planes, the C1–C6 and O3/C8–C10 planes, and the C11–C16 and O6/C18–C20 planes. The corresponding dihedral angles in (III) are 0.67 (16), 15.05 (17), 2.83 (10) and 11.86 (10)°, respectively, between the C1–C6 and O1/C7/O2 planes, the C12–C17 and O4/C18/O5 planes, the C1–C6 and O3/C8–C11 planes, and the C12–C17 and O6/C19–C22 planes.

## Supra­molecular features   

**Figure 2:**
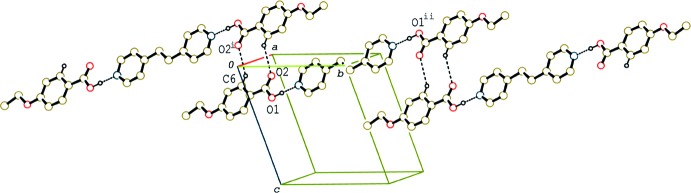
A partial packing diagram of compound (I), showing the tape structure formed by C—H⋯O hydrogen bonds. H atoms not involved in C—H⋯O and O—H⋯N hydrogen bonds (dashed lines) have been omitted [symmetry codes: (i) −*x* + 1, −*y*, −*z*; (ii) −*x*, −*y* + 2, −*z*].

**Figure 3:**
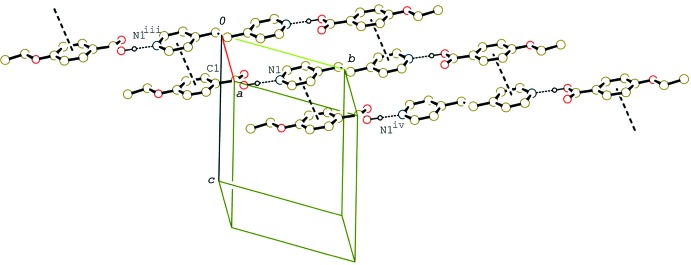
A partial packing diagram of compound (I), showing the column structure formed by π–π stacking inter­actions (dashed lines). H atoms not involved in O—H⋯N hydrogen bonds (dashed lines) have been omitted [symmetry codes: (iii) *x*, *y* − 1, *z*; (iv) *x*, *y* + 1, *z*].

In the crystal of (I), the 2:1 units are linked by a pair of C—H⋯O hydrogen bonds (Table 1 ▸), forming a tape structure along [1
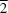
0] (Fig. 2 ▸). In addition, the units are stacked in a column through π–π inter­actions between the acid and base rings along the *b* axis (Fig. 3 ▸). The centroid–centroid distance between the C1–C6 and N1/C10–C14(*x*, *y* − 1, *z*) rings is 3.592 (2) Å.

**Figure 4:**
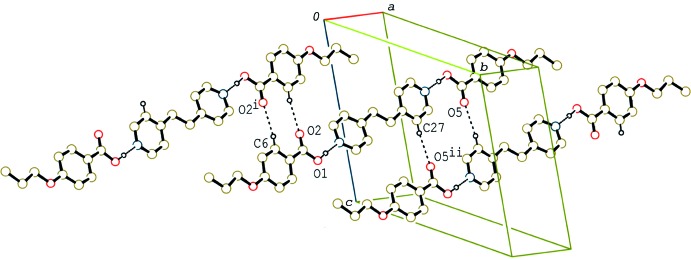
A partial packing diagram of compound (II), showing the tape structure formed by C—H⋯O inter­actions. H atoms not involved in C—H⋯O and O—H⋯N hydrogen bonds (dashed lines) have been omitted [symmetry codes: (i) −*x* − 2, −*y*, −*z* + 1; (ii) −*x* + 1, −*y* + 1, −*z* + 1].

**Figure 5:**
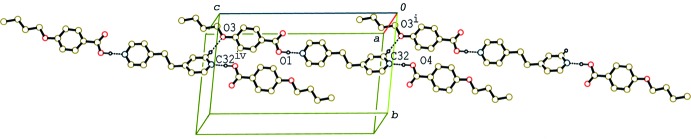
A partial packing diagram of compound (III), showing the tape structure formed by C—H⋯O inter­actions. H atoms not involved in C—H⋯O and O—H⋯N hydrogen bonds (dashed lines) have been omitted [symmetry codes: (i) *x*, *y*, *z* − 1; (iv) *x*, *y*, *z* + 1].

In the crystal of (II) and (III), the 2:1 units are linked by C—H⋯O inter­actions (Tables 2 ▸ and 3 ▸), forming tape structures along [310] (Fig. 4 ▸) and [001] (Fig. 5 ▸), respectively. Between the tapes in (II), a weak π–π inter­action is observed. The centroid–centroid distance between the C11–C16 benzene ring and the N2/C26–C30(*x* + 1, *y*, *z*) pyridine ring is 3.7115 (18) Å. On the other hand, between the tapes in (III) C—H⋯π inter­actions are observed (Table 3 ▸). Although the 2:1 units of the three compounds are arranged in the crystals with their long axes parallel to each other, the distinct layer structure leading to a smectic structure, as observed in 4-*n*-but­oxy­benzoic acid–4,4′-bipyridyl (2/1) (Tabuchi *et al.*, 2015 ▸), is not observed.

## Database survey   

A search of the Cambridge Structural Database (Version 5.36, last update February 2015; Groom & Allen, 2014 ▸) for co-crystals of 1,2-bis­(pyridin-4-yl)ethane with 4-alk­oxy­benzoic acid gave three structures (Mukherjee & Desiraju, 2014 ▸; Aakeröy *et al.*, 2005 ▸). A similar compound, 4-pentyl­benzoic acid–1,2-bis­(pyridin-4-yl)ethane (2/1), was reported to exhibit transitions to liquid-crystalline phases (smectic A at 421.3 K and nematic at 439.6 K) and the mol­ecular motions were investigated by solid-state NMR (Duer *et al.*, 1996 ▸; Clauss *et al.*, 1996 ▸).

## Synthesis and crystallization   

Single crystals of compound (I) were obtained by slow evaporation from an acetone solution (150 ml) of 1,2-bis(pyridin-4-yl)ethane (67 mg) with 4-eth­oxy­benzoic acid (120 mg) at room temperature. Crystals of compounds (II) and (III) were obtained from ethanol solutions of 1,2-bis(pyridin-4-yl)ethane with 4-*n*-propoxybenzoic acid and 4-*n*-but­oxy­benzoic acid, respectively, at room temperature [ethanol solution (150 ml) of 1,2-bis­(pyridin-4-yl)ethane (62 mg) and 4-*n*-propoxybenzoic acid (120 mg) for (II), and ethanol solution (150 ml) of 1,2-bis­(pyridin-4-yl)ethane (57 mg) and 4-*n*-but­oxy­benzoic acid (120 mg) for (III)].

## DSC measurements   

Phase transitions of 4-meth­oxy­benzoic acid–1,2-bis­(pyridin-4-yl)ethane (2/1) and the title three compounds were observed by DSC and the liquid phases were confirmed by polarizing microscopy. DSC measurements were performed by using a PerkinElmer Pyris 1 in the temperature range from 103 K to the melting temperature at a heating rate of 10 K min^−1^. Phase transition temperatures (K) and enthalpies (kJ mol^−1^) determined by DSC are as follows:

4-meth­oxy­benzoic acid–1,2-bis­(pyridin-4-yl)ethane (2/1) 419 (1) [56 (2)] K_1_ → N, 423 (1) [6.3 (13)] N → I;

(I) 353 (3) [4.5 (5)] K_1_ → K_2_, 373 (3) [6.55 (9)] K_2_ → K_3_, 404 (1) [0.89 (15)] K_3_ → K_4_, 421 (1) [49 (3)] K_4_ → N, 434 (1) [11.7 (10)] N → I;

(II) 365 (1) [17.9 (9)] K_1_ → K_2_, 419 (1) [39 (2)] K_2_ → N, 421 (1) [6.3 (2)] N → I;

(III) 339 (2) [4.4 (2)] K_1_ → K_2_, 399 (1) [0.33 (4)] K_2_ → K_3_, 413 (1) [39 (3)] K_3_ → S_A_, 419 (1) [0.74 (13)] S_A_ → N, 424 (1) [9.8 (15)] N → I.

K_i_, S_A_, N and I denote crystal, smectic A, nematic and isotropic phases, respectively.

## Refinement   

Crystal data, data collection and structure refinement details are summarized in Table 4 ▸. For all compounds, C-bound H atoms were positioned geometrically with C—H = 0.93–0.99 Å and were refined as riding with *U*_iso_(H) = 1.2*U*_eq_(C) or 1.5*U*_eq_(methyl C). The O-bound H atoms were located in difference Fourier maps and freely refined [refined O—H = 0.966 (18)–1.03 (3) Å].

## Supplementary Material

Crystal structure: contains datablock(s) I, II, III, General. DOI: 10.1107/S2056989015019349/su5225sup1.cif

Structure factors: contains datablock(s) I. DOI: 10.1107/S2056989015019349/su5225Isup2.hkl

Structure factors: contains datablock(s) II. DOI: 10.1107/S2056989015019349/su5225IIsup3.hkl

Structure factors: contains datablock(s) III. DOI: 10.1107/S2056989015019349/su5225IIIsup4.hkl

Supporting information file. DOI: 10.1107/S2056989015019349/su5225Isup5.cml

Supporting information file. DOI: 10.1107/S2056989015019349/su5225IIsup6.cml

Supporting information file. DOI: 10.1107/S2056989015019349/su5225IIIsup7.cml

CCDC references: 1430875, 1430874, 1430873

Additional supporting information:  crystallographic information; 3D view; checkCIF report

## Figures and Tables

**Table 1:** Hydrogen-bond geometry (Å, °) for (I)

*D*—H⋯*A*	*D*—H	H⋯*A*	*D*⋯*A*	*D*—H⋯*A*
O1—H1⋯N1	0.967 (18)	1.659 (18)	2.6247 (17)	178.0 (14)
C6—H6⋯O2^i^	0.95	2.60	3.406 (2)	144

Symmetry code: (i) 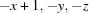.

**Table 2:** Hydrogen-bond geometry (Å, °) for (II)

*D*—H⋯*A*	*D*—H	H⋯*A*	*D*⋯*A*	*D*—H⋯*A*
O1—H1⋯N1	0.966 (18)	1.657 (19)	2.6207 (17)	175.4 (16)
O4—H4⋯N2	1.010 (19)	1.610 (19)	2.6198 (17)	179 (2)
C6—H6⋯O2^i^	0.93	2.55	3.376 (2)	149
C27—H27⋯O5^ii^	0.93	2.52	3.389 (2)	156

Symmetry codes: (i) 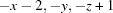; (ii) 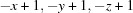.

**Table 3:** Hydrogen-bond geometry (Å, °) for (III) *Cg*1 and *Cg*2 are the centroids of the C1–C6 and C12–C17 rings, respectively.

*D*—H⋯*A*	*D*—H	H⋯*A*	*D*⋯*A*	*D*—H⋯*A*
O1—H1⋯N1	1.02 (2)	1.60 (2)	2.6209 (18)	177 (2)
O4—H4⋯N2	1.03 (3)	1.58 (3)	2.6092 (18)	178.1 (19)
C32—H32⋯O3^i^	0.95	2.57	3.524 (2)	177
C11—H11*A*⋯*Cg*1^ii^	0.98	2.80	3.662 (2)	148
C33—H33*A*⋯*Cg*2^iii^	0.99	2.74	3.598 (2)	145

Symmetry codes: (i) 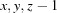; (ii) 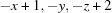; (iii) 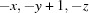.

**Table 4:** Experimental details

	(I)	(II)	(III)
Crystal data			
Chemical formula	2C_9_H_10_O_3_·C_12_H_12_N_2_	2C_10_H_12_O_3_·C_12_H_12_N_2_	2C_11_H_14_O_3_·C_12_H_12_N_2_
*M*_r_	516.57	544.63	572.68
Crystal system, space group	Triclinic, *P* 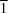	Triclinic, *P* 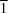	Triclinic, *P* 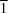
Temperature (K)	93	290	93
*a*, *b*, *c* (Å)	6.967 (3), 9.163 (4), 10.813 (6)	9.121 (3), 12.552 (5), 13.306 (6)	7.702 (2), 10.726 (4), 19.010 (7)
α, β, γ (°)	75.41 (2), 74.97 (2), 77.801 (19)	71.328 (16), 75.076 (18), 89.817 (16)	83.861 (17), 78.794 (16), 73.612 (15)
*V* (Å^3^)	637.3 (6)	1389.2 (9)	1475.5 (9)
*Z*	1	2	2
Radiation type	Mo *K*α	Mo *K*α	Mo *K*α
μ (mm^−1^)	0.09	0.09	0.09
Crystal size (mm)	0.42 × 0.38 × 0.36	0.40 × 0.30 × 0.20	0.40 × 0.20 × 0.10

Data collection			
Diffractometer	Rigaku R-AXIS RAPIDII	Rigaku R-AXIS RAPIDII	Rigaku R-AXIS RAPIDII
Absorption correction	Multi-scan (*ABSCOR*; Higashi, 1995 ▸)	Multi-scan (*ABSCOR*; Higashi, 1995 ▸)	Multi-scan (*ABSCOR*; Higashi, 1995 ▸)
*T*_min_, *T*_max_	0.877, 0.967	0.601, 0.982	0.768, 0.991
No. of measured, independent and observed [*I* > 2σ(*I*)] reflections	6408, 2908, 2628	14034, 6347, 4294	14444, 6670, 5219
*R*_int_	0.014	0.022	0.021
(sin θ/λ)_max_ (Å^−1^)	0.649	0.649	0.649

Refinement			
*R*[*F* ^2^ > 2σ(*F* ^2^)], *wR*(*F* ^2^), *S*	0.038, 0.113, 1.07	0.041, 0.122, 0.97	0.043, 0.129, 1.06
No. of reflections	2908	6347	6670
No. of parameters	177	371	389
H-atom treatment	H atoms treated by a mixture of independent and constrained refinement	H atoms treated by a mixture of independent and constrained refinement	H atoms treated by a mixture of independent and constrained refinement
Δρ_max_, Δρ_min_ (e Å^−3^)	0.25, −0.40	0.18, −0.21	0.22, −0.36

Computer programs: *RAPID-AUTO* (Rigaku, 2006 ▸), *Il Milione* (Burla *et al.*, 2007 ▸), *SHELXL2014*/7 (Sheldrick, 2015 ▸), *ORTEP-3 for Windows* (Farrugia, 2012 ▸), *CrystalStructure* (Rigaku, 2010 ▸) and *PLATON* (Spek, 2009 ▸).

## supplementary crystallographic information

### (I) 4-Ethoxybenzoic acid–1,2-bis(pyridin-4-yl)ethane (2/1) Crystal data

**Table:**

2C_9_H_10_O_3_·C_12_H_12_N_2_	*Z* = 1
*M**_r_* = 516.57	*F*(000) = 274.00
Triclinic, *P*1	*D*_x_ = 1.346 Mg m^−^^3^
*a* = 6.967 (3) Å	Mo *K*α radiation, λ = 0.71075 Å
*b* = 9.163 (4) Å	Cell parameters from 7263 reflections
*c* = 10.813 (6) Å	θ = 3.1–30.0°
α = 75.41 (2)°	µ = 0.09 mm^−^^1^
β = 74.97 (2)°	*T* = 93 K
γ = 77.801 (19)°	Block, colorless
*V* = 637.3 (6) Å^3^	0.42 × 0.38 × 0.36 mm

### (I) 4-Ethoxybenzoic acid–1,2-bis(pyridin-4-yl)ethane (2/1) Data collection

**Table:**

Rigaku R-AXIS RAPIDII diffractometer	2628 reflections with *I* > 2σ(*I*)
Detector resolution: 10.000 pixels mm^-1^	*R*_int_ = 0.014
ω scans	θ_max_ = 27.5°, θ_min_ = 3.1°
Absorption correction: multi-scan (ABSCOR; Higashi, 1995)	*h* = −9→8
*T*_min_ = 0.877, *T*_max_ = 0.967	*k* = −11→11
6408 measured reflections	*l* = −14→14
2908 independent reflections	

### (I) 4-Ethoxybenzoic acid–1,2-bis(pyridin-4-yl)ethane (2/1) Refinement

**Table:**

Refinement on *F*^2^	Primary atom site location: structure-invariant direct methods
Least-squares matrix: full	Secondary atom site location: difference Fourier map
*R*[*F*^2^ > 2σ(*F*^2^)] = 0.038	Hydrogen site location: mixed
*wR*(*F*^2^) = 0.113	H atoms treated by a mixture of independent and constrained refinement
*S* = 1.07	*w* = 1/[σ^2^(*F*_o_^2^) + (0.0808*P*)^2^ + 0.0636*P*] where *P* = (*F*_o_^2^ + 2*F*_c_^2^)/3
2908 reflections	(Δ/σ)_max_ < 0.001
177 parameters	Δρ_max_ = 0.25 e Å^−^^3^
0 restraints	Δρ_min_ = −0.40 e Å^−^^3^

### (I) 4-Ethoxybenzoic acid–1,2-bis(pyridin-4-yl)ethane (2/1) Special details

**Table:**

Geometry. All e.s.d.'s (except the e.s.d. in the dihedral angle between two l.s. planes) are estimated using the full covariance matrix. The cell e.s.d.'s are taken into account individually in the estimation of e.s.d.'s in distances, angles and torsion angles; correlations between e.s.d.'s in cell parameters are only used when they are defined by crystal symmetry. An approximate (isotropic) treatment of cell e.s.d.'s is used for estimating e.s.d.'s involving l.s. planes.
Refinement. Reflections were merged by *SHELXL* according to the crystal class for the calculation of statistics and refinement.

### (I) 4-Ethoxybenzoic acid–1,2-bis(pyridin-4-yl)ethane (2/1) Fractional atomic coordinates and isotropic or equivalent isotropic displacement parameters (Å^2^)

**Table:**

	*x*	*y*	*z*	*U*_iso_*/*U*_eq_	
O1	0.08950 (10)	0.17801 (7)	0.27364 (6)	0.01773 (17)	
O2	0.36783 (10)	0.14762 (7)	0.11743 (7)	0.02284 (18)	
O3	0.30648 (10)	−0.53714 (7)	0.43129 (6)	0.01635 (17)	
N1	0.08580 (11)	0.46999 (8)	0.17005 (7)	0.01554 (18)	
C1	0.25803 (12)	−0.07314 (9)	0.26753 (8)	0.01328 (19)	
C2	0.12341 (13)	−0.13188 (10)	0.38081 (8)	0.01439 (19)	
H2	0.0175	−0.0645	0.4223	0.017*	
C3	0.14273 (13)	−0.28698 (9)	0.43308 (8)	0.01467 (19)	
H3	0.0511	−0.3258	0.5104	0.018*	
C4	0.29770 (13)	−0.38638 (9)	0.37165 (8)	0.01337 (19)	
C5	0.42960 (12)	−0.32990 (9)	0.25669 (8)	0.01389 (19)	
H5	0.5326	−0.3976	0.2135	0.017*	
C6	0.40911 (12)	−0.17344 (9)	0.20570 (8)	0.01382 (19)	
H6	0.4994	−0.1346	0.1277	0.017*	
C7	0.24541 (13)	0.09388 (10)	0.21156 (8)	0.01440 (19)	
C8	0.46043 (13)	−0.64340 (10)	0.36946 (9)	0.0164 (2)	
H8A	0.5947	−0.6172	0.3609	0.020*	
H8B	0.4421	−0.6395	0.2808	0.020*	
C9	0.44480 (14)	−0.80100 (10)	0.45311 (10)	0.0199 (2)	
H9A	0.4609	−0.8032	0.5410	0.030*	
H9B	0.5506	−0.8753	0.4136	0.030*	
H9C	0.3128	−0.8269	0.4591	0.030*	
C10	0.21117 (14)	0.51152 (10)	0.05431 (9)	0.0174 (2)	
H10	0.3031	0.4343	0.0159	0.021*	
C11	0.21180 (13)	0.66206 (10)	−0.01116 (8)	0.0168 (2)	
H11	0.3038	0.6865	−0.0924	0.020*	
C12	0.07739 (13)	0.77805 (9)	0.04216 (8)	0.01326 (19)	
C13	−0.05253 (12)	0.73458 (9)	0.16120 (8)	0.01421 (19)	
H13	−0.1479	0.8093	0.2010	0.017*	
C14	−0.04226 (12)	0.58080 (9)	0.22195 (8)	0.01511 (19)	
H14	−0.1305	0.5533	0.3043	0.018*	
C15	0.08332 (12)	0.94192 (9)	−0.02916 (8)	0.01380 (19)	
H15A	0.0760	0.9504	−0.1210	0.017*	
H15B	0.2145	0.9686	−0.0308	0.017*	
H1	0.091 (2)	0.285 (2)	0.2343 (17)	0.060 (5)*	

### (I) 4-Ethoxybenzoic acid–1,2-bis(pyridin-4-yl)ethane (2/1) Atomic displacement parameters (Å^2^)

**Table:**

	*U*^11^	*U*^22^	*U*^33^	*U*^12^	*U*^13^	*U*^23^
O1	0.0214 (3)	0.0103 (3)	0.0183 (3)	−0.0011 (2)	−0.0008 (2)	−0.0020 (2)
O2	0.0258 (4)	0.0134 (3)	0.0224 (4)	−0.0043 (3)	0.0035 (3)	0.0005 (2)
O3	0.0206 (3)	0.0087 (3)	0.0158 (3)	−0.0015 (2)	−0.0006 (2)	0.0005 (2)
N1	0.0183 (4)	0.0113 (3)	0.0182 (4)	−0.0028 (3)	−0.0076 (3)	−0.0011 (3)
C1	0.0161 (4)	0.0112 (4)	0.0135 (4)	−0.0030 (3)	−0.0053 (3)	−0.0014 (3)
C2	0.0165 (4)	0.0128 (4)	0.0139 (4)	−0.0016 (3)	−0.0027 (3)	−0.0039 (3)
C3	0.0171 (4)	0.0138 (4)	0.0119 (4)	−0.0037 (3)	−0.0008 (3)	−0.0018 (3)
C4	0.0160 (4)	0.0105 (4)	0.0139 (4)	−0.0026 (3)	−0.0053 (3)	−0.0007 (3)
C5	0.0141 (4)	0.0122 (4)	0.0150 (4)	−0.0006 (3)	−0.0035 (3)	−0.0030 (3)
C6	0.0138 (4)	0.0143 (4)	0.0125 (4)	−0.0037 (3)	−0.0022 (3)	−0.0008 (3)
C7	0.0168 (4)	0.0128 (4)	0.0144 (4)	−0.0027 (3)	−0.0048 (3)	−0.0025 (3)
C8	0.0167 (4)	0.0116 (4)	0.0189 (4)	0.0000 (3)	−0.0035 (3)	−0.0021 (3)
C9	0.0234 (4)	0.0114 (4)	0.0241 (5)	−0.0029 (3)	−0.0075 (4)	0.0004 (3)
C10	0.0220 (4)	0.0125 (4)	0.0175 (4)	−0.0009 (3)	−0.0050 (3)	−0.0037 (3)
C11	0.0205 (4)	0.0134 (4)	0.0145 (4)	−0.0024 (3)	−0.0020 (3)	−0.0016 (3)
C12	0.0147 (4)	0.0115 (4)	0.0151 (4)	−0.0029 (3)	−0.0070 (3)	−0.0009 (3)
C13	0.0132 (4)	0.0116 (4)	0.0169 (4)	−0.0010 (3)	−0.0035 (3)	−0.0019 (3)
C14	0.0152 (4)	0.0128 (4)	0.0163 (4)	−0.0036 (3)	−0.0042 (3)	0.0008 (3)
C15	0.0152 (4)	0.0106 (4)	0.0142 (4)	−0.0026 (3)	−0.0031 (3)	0.0002 (3)

### (I) 4-Ethoxybenzoic acid–1,2-bis(pyridin-4-yl)ethane (2/1) Geometric parameters (Å, º)

**Table:**

O1—C7	1.3242 (11)	C8—C9	1.5076 (13)
O1—H1	0.970 (19)	C8—H8A	0.9900
O2—C7	1.2169 (12)	C8—H8B	0.9900
O3—C4	1.3653 (11)	C9—H9A	0.9800
O3—C8	1.4353 (11)	C9—H9B	0.9800
N1—C14	1.3365 (12)	C9—H9C	0.9800
N1—C10	1.3445 (13)	C10—C11	1.3831 (13)
C1—C6	1.3912 (12)	C10—H10	0.9500
C1—C2	1.3983 (13)	C11—C12	1.3954 (12)
C1—C7	1.4900 (13)	C11—H11	0.9500
C2—C3	1.3825 (13)	C12—C13	1.3874 (14)
C2—H2	0.9500	C12—C15	1.5091 (13)
C3—C4	1.3978 (12)	C13—C14	1.3920 (12)
C3—H3	0.9500	C13—H13	0.9500
C4—C5	1.3936 (14)	C14—H14	0.9500
C5—C6	1.3913 (12)	C15—C15^i^	1.5215 (16)
C5—H5	0.9500	C15—H15A	0.9900
C6—H6	0.9500	C15—H15B	0.9900
			
C7—O1—H1	110.2 (10)	H8A—C8—H8B	108.4
C4—O3—C8	117.24 (7)	C8—C9—H9A	109.5
C14—N1—C10	117.48 (8)	C8—C9—H9B	109.5
C6—C1—C2	119.07 (8)	H9A—C9—H9B	109.5
C6—C1—C7	118.99 (8)	C8—C9—H9C	109.5
C2—C1—C7	121.94 (8)	H9A—C9—H9C	109.5
C3—C2—C1	120.72 (8)	H9B—C9—H9C	109.5
C3—C2—H2	119.6	N1—C10—C11	122.73 (8)
C1—C2—H2	119.6	N1—C10—H10	118.6
C2—C3—C4	119.69 (8)	C11—C10—H10	118.6
C2—C3—H3	120.2	C10—C11—C12	120.01 (9)
C4—C3—H3	120.2	C10—C11—H11	120.0
O3—C4—C5	124.27 (8)	C12—C11—H11	120.0
O3—C4—C3	115.52 (8)	C13—C12—C11	117.06 (8)
C5—C4—C3	120.21 (8)	C13—C12—C15	123.64 (8)
C6—C5—C4	119.43 (8)	C11—C12—C15	119.28 (8)
C6—C5—H5	120.3	C12—C13—C14	119.55 (8)
C4—C5—H5	120.3	C12—C13—H13	120.2
C1—C6—C5	120.84 (8)	C14—C13—H13	120.2
C1—C6—H6	119.6	N1—C14—C13	123.16 (8)
C5—C6—H6	119.6	N1—C14—H14	118.4
O2—C7—O1	123.25 (9)	C13—C14—H14	118.4
O2—C7—C1	122.84 (8)	C12—C15—C15^i^	115.14 (9)
O1—C7—C1	113.92 (8)	C12—C15—H15A	108.5
O3—C8—C9	108.07 (8)	C15^i^—C15—H15A	108.5
O3—C8—H8A	110.1	C12—C15—H15B	108.5
C9—C8—H8A	110.1	C15^i^—C15—H15B	108.5
O3—C8—H8B	110.1	H15A—C15—H15B	107.5
C9—C8—H8B	110.1		
			
C6—C1—C2—C3	1.73 (13)	C6—C1—C7—O1	176.33 (7)
C7—C1—C2—C3	−177.82 (7)	C2—C1—C7—O1	−4.12 (12)
C1—C2—C3—C4	−0.40 (13)	C4—O3—C8—C9	178.74 (7)
C8—O3—C4—C5	−0.98 (12)	C14—N1—C10—C11	0.07 (13)
C8—O3—C4—C3	178.48 (7)	N1—C10—C11—C12	−0.53 (14)
C2—C3—C4—O3	179.15 (7)	C10—C11—C12—C13	0.12 (13)
C2—C3—C4—C5	−1.37 (13)	C10—C11—C12—C15	178.65 (8)
O3—C4—C5—C6	−178.79 (7)	C11—C12—C13—C14	0.71 (12)
C3—C4—C5—C6	1.78 (13)	C15—C12—C13—C14	−177.75 (7)
C2—C1—C6—C5	−1.31 (13)	C10—N1—C14—C13	0.83 (12)
C7—C1—C6—C5	178.25 (7)	C12—C13—C14—N1	−1.24 (13)
C4—C5—C6—C1	−0.43 (13)	C13—C12—C15—C15^i^	−7.83 (14)
C6—C1—C7—O2	−3.77 (13)	C11—C12—C15—C15^i^	173.74 (8)
C2—C1—C7—O2	175.77 (8)		

Symmetry code: (i) −*x*, −*y*+2, −*z*.

### (I) 4-Ethoxybenzoic acid–1,2-bis(pyridin-4-yl)ethane (2/1) Hydrogen-bond geometry (Å, º)

**Table:**

*D*—H···*A*	*D*—H	H···*A*	*D*···*A*	*D*—H···*A*
O1—H1···N1	0.967 (18)	1.659 (18)	2.6247 (17)	178.0 (14)
C6—H6···O2^ii^	0.95	2.60	3.406 (2)	144

Symmetry code: (ii) −*x*+1, −*y*, −*z*.

### (II) 4-n-Propoxybenzoic acid–1,2-bis(pyridin-4-yl)ethane (2/1) Crystal data

**Table:**

2C_10_H_12_O_3_·C_12_H_12_N_2_	*Z* = 2
*M**_r_* = 544.63	*F*(000) = 580.00
Triclinic, *P*1	*D*_x_ = 1.302 Mg m^−^^3^
*a* = 9.121 (3) Å	Mo *K*α radiation, λ = 0.71075 Å
*b* = 12.552 (5) Å	Cell parameters from 12886 reflections
*c* = 13.306 (6) Å	θ = 3.0–30.1°
α = 71.328 (16)°	µ = 0.09 mm^−^^1^
β = 75.076 (18)°	*T* = 290 K
γ = 89.817 (16)°	Block, colorless
*V* = 1389.2 (9) Å^3^	0.40 × 0.30 × 0.20 mm

### (II) 4-n-Propoxybenzoic acid–1,2-bis(pyridin-4-yl)ethane (2/1) Data collection

**Table:**

Rigaku R-AXIS RAPIDII diffractometer	4294 reflections with *I* > 2σ(*I*)
Detector resolution: 10.000 pixels mm^-1^	*R*_int_ = 0.022
ω scans	θ_max_ = 27.5°, θ_min_ = 3.0°
Absorption correction: multi-scan (ABSCOR; Higashi, 1995)	*h* = −11→11
*T*_min_ = 0.601, *T*_max_ = 0.982	*k* = −16→16
14034 measured reflections	*l* = −17→17
6347 independent reflections	

### (II) 4-n-Propoxybenzoic acid–1,2-bis(pyridin-4-yl)ethane (2/1) Refinement

**Table:**

Refinement on *F*^2^	Primary atom site location: structure-invariant direct methods
Least-squares matrix: full	Secondary atom site location: difference Fourier map
*R*[*F*^2^ > 2σ(*F*^2^)] = 0.041	Hydrogen site location: mixed
*wR*(*F*^2^) = 0.122	H atoms treated by a mixture of independent and constrained refinement
*S* = 0.97	*w* = 1/[σ^2^(*F*_o_^2^) + (0.0771*P*)^2^] where *P* = (*F*_o_^2^ + 2*F*_c_^2^)/3
6347 reflections	(Δ/σ)_max_ = 0.001
371 parameters	Δρ_max_ = 0.18 e Å^−^^3^
0 restraints	Δρ_min_ = −0.21 e Å^−^^3^

### (II) 4-n-Propoxybenzoic acid–1,2-bis(pyridin-4-yl)ethane (2/1) Special details

**Table:**

Geometry. All e.s.d.'s (except the e.s.d. in the dihedral angle between two l.s. planes) are estimated using the full covariance matrix. The cell e.s.d.'s are taken into account individually in the estimation of e.s.d.'s in distances, angles and torsion angles; correlations between e.s.d.'s in cell parameters are only used when they are defined by crystal symmetry. An approximate (isotropic) treatment of cell e.s.d.'s is used for estimating e.s.d.'s involving l.s. planes.
Refinement. Reflections were merged by *SHELXL* according to the crystal class for the calculation of statistics and refinement.

### (II) 4-n-Propoxybenzoic acid–1,2-bis(pyridin-4-yl)ethane (2/1) Fractional atomic coordinates and isotropic or equivalent isotropic displacement parameters (Å^2^)

**Table:**

	*x*	*y*	*z*	*U*_iso_*/*U*_eq_	
O1	−0.86106 (9)	0.14285 (8)	0.69416 (7)	0.0513 (2)	
O2	−0.85639 (9)	0.04844 (8)	0.57825 (7)	0.0572 (3)	
O3	−1.54698 (8)	−0.05094 (7)	0.86644 (7)	0.0468 (2)	
O4	0.77073 (9)	0.45876 (7)	0.19717 (7)	0.0492 (2)	
O5	0.76146 (9)	0.54551 (8)	0.31997 (7)	0.0581 (3)	
O6	1.46050 (9)	0.64460 (7)	0.05092 (6)	0.0470 (2)	
N1	−0.57003 (10)	0.19071 (8)	0.59325 (8)	0.0432 (2)	
N2	0.47841 (10)	0.40765 (8)	0.29105 (8)	0.0438 (2)	
C1	−1.08880 (11)	0.04396 (9)	0.71057 (8)	0.0356 (2)	
C2	−1.16087 (12)	0.06518 (9)	0.80729 (9)	0.0386 (2)	
H2	−1.1057	0.1020	0.8382	0.046*	
C3	−1.31324 (12)	0.03204 (9)	0.85748 (9)	0.0407 (3)	
H3	−1.3601	0.0457	0.9225	0.049*	
C4	−1.39693 (12)	−0.02169 (9)	0.81125 (9)	0.0380 (2)	
C5	−1.32604 (12)	−0.04396 (10)	0.71520 (9)	0.0407 (3)	
H5	−1.3811	−0.0807	0.6843	0.049*	
C6	−1.17302 (12)	−0.01094 (9)	0.66615 (9)	0.0399 (2)	
H6	−1.1256	−0.0259	0.6019	0.048*	
C7	−0.92513 (12)	0.07793 (9)	0.65432 (9)	0.0384 (2)	
C8	−1.63612 (12)	−0.10691 (10)	0.82118 (10)	0.0449 (3)	
H8A	−1.5929	−0.1764	0.8164	0.054*	
H8B	−1.6348	−0.0591	0.7474	0.054*	
C9	−1.79706 (13)	−0.13286 (11)	0.89263 (10)	0.0484 (3)	
H9A	−1.8427	−0.0631	0.8933	0.058*	
H9B	−1.7979	−0.1763	0.9676	0.058*	
C10	−1.88892 (14)	−0.19964 (12)	0.84880 (11)	0.0557 (3)	
H10A	−1.8425	−0.2680	0.8471	0.084*	
H10B	−1.8912	−0.1552	0.7756	0.084*	
H10C	−1.9910	−0.2178	0.8960	0.084*	
C11	0.99847 (12)	0.55101 (9)	0.19183 (9)	0.0372 (2)	
C12	1.08279 (12)	0.51427 (9)	0.10802 (9)	0.0407 (3)	
H12	1.0350	0.4672	0.0823	0.049*	
C13	1.23598 (13)	0.54654 (10)	0.06260 (9)	0.0426 (3)	
H13	1.2907	0.5217	0.0062	0.051*	
C14	1.30898 (12)	0.61617 (9)	0.10086 (9)	0.0394 (2)	
C15	1.22638 (12)	0.65343 (10)	0.18464 (9)	0.0436 (3)	
H15	1.2744	0.6997	0.2111	0.052*	
C16	1.07223 (13)	0.62128 (10)	0.22845 (9)	0.0437 (3)	
H16	1.0169	0.6474	0.2837	0.052*	
C17	0.83294 (12)	0.51878 (9)	0.24279 (9)	0.0400 (2)	
C18	1.53914 (13)	0.70883 (10)	0.09605 (10)	0.0467 (3)	
H18A	1.5325	0.6659	0.1722	0.056*	
H18B	1.4910	0.7782	0.0944	0.056*	
C19	1.70299 (13)	0.73646 (11)	0.03172 (10)	0.0467 (3)	
H19A	1.7104	0.7790	−0.0447	0.056*	
H19B	1.7522	0.6674	0.0342	0.056*	
C20	1.78187 (14)	0.80588 (11)	0.08115 (11)	0.0557 (3)	
H20A	1.7796	0.7617	0.1553	0.084*	
H20B	1.7300	0.8725	0.0813	0.084*	
H20C	1.8857	0.8271	0.0379	0.084*	
C21	−0.50433 (13)	0.14613 (10)	0.51637 (10)	0.0448 (3)	
H21	−0.5642	0.0987	0.4987	0.054*	
C22	−0.35180 (12)	0.16703 (10)	0.46180 (10)	0.0443 (3)	
H22	−0.3108	0.1336	0.4088	0.053*	
C23	−0.25902 (12)	0.23753 (9)	0.48532 (9)	0.0369 (2)	
C24	−0.32816 (13)	0.28490 (11)	0.56375 (10)	0.0494 (3)	
H24	−0.2715	0.3339	0.5819	0.059*	
C25	−0.48225 (13)	0.25931 (12)	0.61548 (10)	0.0543 (3)	
H25	−0.5265	0.2919	0.6685	0.065*	
C26	0.41740 (13)	0.43030 (11)	0.38378 (10)	0.0474 (3)	
H26	0.4803	0.4649	0.4114	0.057*	
C27	0.26572 (13)	0.40488 (10)	0.44034 (10)	0.0459 (3)	
H27	0.2284	0.4220	0.5048	0.055*	
C28	0.16865 (11)	0.35392 (9)	0.40132 (9)	0.0376 (2)	
C29	0.23253 (12)	0.32884 (10)	0.30641 (9)	0.0424 (3)	
H29	0.1728	0.2926	0.2780	0.051*	
C30	0.38599 (13)	0.35788 (10)	0.25382 (10)	0.0453 (3)	
H30	0.4264	0.3417	0.1892	0.054*	
C31	−0.09183 (12)	0.25894 (10)	0.42528 (9)	0.0401 (3)	
H31A	−0.0818	0.2946	0.3472	0.048*	
H31B	−0.0489	0.1867	0.4347	0.048*	
C32	0.00142 (12)	0.33167 (10)	0.46139 (9)	0.0406 (3)	
H32A	−0.0420	0.4037	0.4524	0.049*	
H32B	−0.0084	0.2958	0.5394	0.049*	
H1	−0.755 (2)	0.1609 (15)	0.6535 (14)	0.098 (6)*	
H4	0.658 (2)	0.4399 (16)	0.2335 (15)	0.106 (6)*	

### (II) 4-n-Propoxybenzoic acid–1,2-bis(pyridin-4-yl)ethane (2/1) Atomic displacement parameters (Å^2^)

**Table:**

	*U*^11^	*U*^22^	*U*^33^	*U*^12^	*U*^13^	*U*^23^
O1	0.0343 (4)	0.0726 (6)	0.0542 (5)	−0.0057 (4)	−0.0016 (4)	−0.0386 (5)
O2	0.0386 (4)	0.0833 (6)	0.0575 (5)	−0.0022 (4)	0.0016 (4)	−0.0451 (5)
O3	0.0326 (4)	0.0641 (5)	0.0447 (5)	−0.0036 (4)	−0.0004 (3)	−0.0273 (4)
O4	0.0359 (4)	0.0656 (5)	0.0546 (5)	−0.0020 (4)	−0.0107 (4)	−0.0324 (4)
O5	0.0434 (5)	0.0806 (6)	0.0562 (5)	−0.0068 (4)	−0.0011 (4)	−0.0401 (5)
O6	0.0368 (4)	0.0590 (5)	0.0484 (5)	−0.0050 (4)	−0.0037 (3)	−0.0281 (4)
N1	0.0339 (5)	0.0533 (6)	0.0421 (5)	−0.0010 (4)	−0.0052 (4)	−0.0194 (4)
N2	0.0337 (5)	0.0465 (5)	0.0497 (6)	0.0016 (4)	−0.0118 (4)	−0.0135 (5)
C1	0.0344 (5)	0.0373 (5)	0.0354 (6)	0.0066 (4)	−0.0080 (4)	−0.0137 (5)
C2	0.0375 (5)	0.0444 (6)	0.0388 (6)	0.0037 (5)	−0.0096 (4)	−0.0210 (5)
C3	0.0398 (6)	0.0493 (6)	0.0350 (6)	0.0048 (5)	−0.0038 (4)	−0.0217 (5)
C4	0.0341 (5)	0.0408 (6)	0.0371 (6)	0.0033 (4)	−0.0051 (4)	−0.0139 (5)
C5	0.0367 (5)	0.0497 (6)	0.0397 (6)	0.0000 (5)	−0.0079 (4)	−0.0222 (5)
C6	0.0386 (6)	0.0479 (6)	0.0354 (6)	0.0043 (5)	−0.0048 (4)	−0.0209 (5)
C7	0.0344 (5)	0.0447 (6)	0.0374 (6)	0.0045 (5)	−0.0071 (4)	−0.0171 (5)
C8	0.0373 (6)	0.0527 (7)	0.0468 (7)	−0.0009 (5)	−0.0063 (5)	−0.0230 (5)
C9	0.0389 (6)	0.0548 (7)	0.0466 (7)	−0.0028 (5)	−0.0011 (5)	−0.0184 (6)
C10	0.0407 (6)	0.0621 (8)	0.0621 (8)	−0.0084 (6)	−0.0018 (6)	−0.0270 (7)
C11	0.0370 (5)	0.0407 (6)	0.0357 (6)	0.0032 (4)	−0.0124 (4)	−0.0130 (5)
C12	0.0423 (6)	0.0460 (6)	0.0399 (6)	0.0002 (5)	−0.0151 (5)	−0.0194 (5)
C13	0.0438 (6)	0.0503 (6)	0.0383 (6)	0.0030 (5)	−0.0088 (5)	−0.0224 (5)
C14	0.0380 (5)	0.0427 (6)	0.0378 (6)	0.0026 (5)	−0.0096 (4)	−0.0141 (5)
C15	0.0401 (6)	0.0494 (6)	0.0469 (6)	−0.0026 (5)	−0.0100 (5)	−0.0251 (5)
C16	0.0429 (6)	0.0510 (7)	0.0413 (6)	0.0032 (5)	−0.0073 (5)	−0.0240 (5)
C17	0.0390 (6)	0.0432 (6)	0.0395 (6)	0.0040 (5)	−0.0123 (5)	−0.0149 (5)
C18	0.0424 (6)	0.0506 (7)	0.0512 (7)	−0.0017 (5)	−0.0083 (5)	−0.0256 (6)
C19	0.0397 (6)	0.0505 (7)	0.0487 (7)	−0.0024 (5)	−0.0066 (5)	−0.0191 (5)
C20	0.0434 (6)	0.0641 (8)	0.0609 (8)	−0.0070 (6)	−0.0073 (6)	−0.0276 (7)
C21	0.0378 (6)	0.0512 (7)	0.0484 (7)	−0.0026 (5)	−0.0084 (5)	−0.0229 (5)
C22	0.0381 (6)	0.0529 (7)	0.0457 (6)	0.0014 (5)	−0.0045 (5)	−0.0266 (5)
C23	0.0332 (5)	0.0411 (6)	0.0350 (5)	0.0034 (4)	−0.0078 (4)	−0.0119 (5)
C24	0.0387 (6)	0.0653 (8)	0.0511 (7)	−0.0061 (5)	−0.0058 (5)	−0.0332 (6)
C25	0.0408 (6)	0.0771 (9)	0.0513 (7)	−0.0030 (6)	−0.0006 (5)	−0.0388 (7)
C26	0.0393 (6)	0.0556 (7)	0.0502 (7)	−0.0022 (5)	−0.0164 (5)	−0.0177 (6)
C27	0.0408 (6)	0.0571 (7)	0.0428 (6)	0.0000 (5)	−0.0119 (5)	−0.0202 (5)
C28	0.0334 (5)	0.0388 (6)	0.0388 (6)	0.0033 (4)	−0.0103 (4)	−0.0101 (5)
C29	0.0356 (5)	0.0474 (6)	0.0486 (7)	0.0004 (5)	−0.0120 (5)	−0.0210 (5)
C30	0.0376 (6)	0.0494 (7)	0.0489 (7)	0.0036 (5)	−0.0072 (5)	−0.0200 (5)
C31	0.0322 (5)	0.0482 (6)	0.0397 (6)	0.0003 (5)	−0.0050 (4)	−0.0178 (5)
C32	0.0342 (5)	0.0483 (6)	0.0398 (6)	0.0008 (5)	−0.0079 (4)	−0.0169 (5)

### (II) 4-n-Propoxybenzoic acid–1,2-bis(pyridin-4-yl)ethane (2/1) Geometric parameters (Å, º)

**Table:**

O1—C7	1.3136 (14)	C13—C14	1.3891 (15)
O1—H1	0.963 (19)	C13—H13	0.9300
O2—C7	1.2108 (14)	C14—C15	1.3885 (16)
O3—C4	1.3638 (13)	C15—C16	1.3821 (16)
O3—C8	1.4297 (13)	C15—H15	0.9300
O4—C17	1.3157 (14)	C16—H16	0.9300
O4—H4	1.01 (2)	C18—C19	1.4969 (16)
O5—C17	1.2110 (14)	C18—H18A	0.9700
O6—C14	1.3628 (13)	C18—H18B	0.9700
O6—C18	1.4355 (13)	C19—C20	1.5245 (16)
N1—C25	1.3264 (15)	C19—H19A	0.9700
N1—C21	1.3294 (15)	C19—H19B	0.9700
N2—C30	1.3277 (15)	C20—H20A	0.9600
N2—C26	1.3352 (16)	C20—H20B	0.9600
C1—C6	1.3828 (15)	C20—H20C	0.9600
C1—C2	1.3935 (15)	C21—C22	1.3754 (16)
C1—C7	1.4815 (15)	C21—H21	0.9300
C2—C3	1.3778 (15)	C22—C23	1.3841 (15)
C2—H2	0.9300	C22—H22	0.9300
C3—C4	1.3881 (15)	C23—C24	1.3770 (16)
C3—H3	0.9300	C23—C31	1.5079 (15)
C4—C5	1.3897 (16)	C24—C25	1.3840 (16)
C5—C6	1.3810 (15)	C24—H24	0.9300
C5—H5	0.9300	C25—H25	0.9300
C6—H6	0.9300	C26—C27	1.3766 (17)
C8—C9	1.5002 (16)	C26—H26	0.9300
C8—H8A	0.9700	C27—C28	1.3841 (15)
C8—H8B	0.9700	C27—H27	0.9300
C9—C10	1.5211 (16)	C28—C29	1.3812 (16)
C9—H9A	0.9700	C28—C32	1.5084 (15)
C9—H9B	0.9700	C29—C30	1.3846 (16)
C10—H10A	0.9600	C29—H29	0.9300
C10—H10B	0.9600	C30—H30	0.9300
C10—H10C	0.9600	C31—C32	1.5113 (16)
C11—C16	1.3842 (15)	C31—H31A	0.9700
C11—C12	1.3903 (16)	C31—H31B	0.9700
C11—C17	1.4846 (16)	C32—H32A	0.9700
C12—C13	1.3761 (16)	C32—H32B	0.9700
C12—H12	0.9300		
			
C7—O1—H1	109.0 (11)	O5—C17—O4	122.94 (10)
C4—O3—C8	117.37 (9)	O5—C17—C11	122.69 (10)
C17—O4—H4	111.7 (11)	O4—C17—C11	114.38 (10)
C14—O6—C18	116.48 (9)	O6—C18—C19	110.74 (10)
C25—N1—C21	117.15 (10)	O6—C18—H18A	109.5
C30—N2—C26	117.12 (10)	C19—C18—H18A	109.5
C6—C1—C2	118.76 (10)	O6—C18—H18B	109.5
C6—C1—C7	119.13 (10)	C19—C18—H18B	109.5
C2—C1—C7	122.11 (10)	H18A—C18—H18B	108.1
C3—C2—C1	120.48 (10)	C18—C19—C20	109.14 (10)
C3—C2—H2	119.8	C18—C19—H19A	109.9
C1—C2—H2	119.8	C20—C19—H19A	109.9
C2—C3—C4	120.17 (10)	C18—C19—H19B	109.9
C2—C3—H3	119.9	C20—C19—H19B	109.9
C4—C3—H3	119.9	H19A—C19—H19B	108.3
O3—C4—C3	116.02 (10)	C19—C20—H20A	109.5
O3—C4—C5	124.12 (10)	C19—C20—H20B	109.5
C3—C4—C5	119.85 (10)	H20A—C20—H20B	109.5
C6—C5—C4	119.36 (10)	C19—C20—H20C	109.5
C6—C5—H5	120.3	H20A—C20—H20C	109.5
C4—C5—H5	120.3	H20B—C20—H20C	109.5
C5—C6—C1	121.37 (10)	N1—C21—C22	122.90 (10)
C5—C6—H6	119.3	N1—C21—H21	118.6
C1—C6—H6	119.3	C22—C21—H21	118.6
O2—C7—O1	122.92 (10)	C21—C22—C23	120.34 (11)
O2—C7—C1	122.94 (10)	C21—C22—H22	119.8
O1—C7—C1	114.14 (10)	C23—C22—H22	119.8
O3—C8—C9	109.94 (10)	C24—C23—C22	116.51 (10)
O3—C8—H8A	109.7	C24—C23—C31	123.83 (10)
C9—C8—H8A	109.7	C22—C23—C31	119.66 (10)
O3—C8—H8B	109.7	C23—C24—C25	119.76 (11)
C9—C8—H8B	109.7	C23—C24—H24	120.1
H8A—C8—H8B	108.2	C25—C24—H24	120.1
C8—C9—C10	109.84 (10)	N1—C25—C24	123.33 (11)
C8—C9—H9A	109.7	N1—C25—H25	118.3
C10—C9—H9A	109.7	C24—C25—H25	118.3
C8—C9—H9B	109.7	N2—C26—C27	123.04 (11)
C10—C9—H9B	109.7	N2—C26—H26	118.5
H9A—C9—H9B	108.2	C27—C26—H26	118.5
C9—C10—H10A	109.5	C26—C27—C28	120.05 (11)
C9—C10—H10B	109.5	C26—C27—H27	120.0
H10A—C10—H10B	109.5	C28—C27—H27	120.0
C9—C10—H10C	109.5	C29—C28—C27	116.80 (10)
H10A—C10—H10C	109.5	C29—C28—C32	123.87 (10)
H10B—C10—H10C	109.5	C27—C28—C32	119.32 (10)
C16—C11—C12	118.39 (10)	C28—C29—C30	119.67 (10)
C16—C11—C17	118.83 (10)	C28—C29—H29	120.2
C12—C11—C17	122.78 (10)	C30—C29—H29	120.2
C13—C12—C11	120.97 (10)	N2—C30—C29	123.30 (11)
C13—C12—H12	119.5	N2—C30—H30	118.3
C11—C12—H12	119.5	C29—C30—H30	118.3
C12—C13—C14	120.07 (10)	C23—C31—C32	115.61 (10)
C12—C13—H13	120.0	C23—C31—H31A	108.4
C14—C13—H13	120.0	C32—C31—H31A	108.4
O6—C14—C15	124.08 (10)	C23—C31—H31B	108.4
O6—C14—C13	116.27 (10)	C32—C31—H31B	108.4
C15—C14—C13	119.64 (10)	H31A—C31—H31B	107.4
C16—C15—C14	119.53 (10)	C28—C32—C31	115.92 (10)
C16—C15—H15	120.2	C28—C32—H32A	108.3
C14—C15—H15	120.2	C31—C32—H32A	108.3
C15—C16—C11	121.38 (11)	C28—C32—H32B	108.3
C15—C16—H16	119.3	C31—C32—H32B	108.3
C11—C16—H16	119.3	H32A—C32—H32B	107.4
			
C6—C1—C2—C3	−0.05 (16)	C17—C11—C16—C15	179.52 (10)
C7—C1—C2—C3	−179.81 (10)	C16—C11—C17—O5	−4.84 (17)
C1—C2—C3—C4	−0.79 (17)	C12—C11—C17—O5	175.67 (11)
C8—O3—C4—C3	−179.71 (10)	C16—C11—C17—O4	175.24 (9)
C8—O3—C4—C5	−0.69 (16)	C12—C11—C17—O4	−4.25 (16)
C2—C3—C4—O3	−179.73 (10)	C14—O6—C18—C19	−178.58 (9)
C2—C3—C4—C5	1.21 (17)	O6—C18—C19—C20	179.25 (10)
O3—C4—C5—C6	−179.77 (10)	C25—N1—C21—C22	0.98 (18)
C3—C4—C5—C6	−0.78 (17)	N1—C21—C22—C23	−0.30 (19)
C4—C5—C6—C1	−0.06 (17)	C21—C22—C23—C24	−0.73 (18)
C2—C1—C6—C5	0.48 (17)	C21—C22—C23—C31	179.59 (10)
C7—C1—C6—C5	−179.76 (10)	C22—C23—C24—C25	1.05 (18)
C6—C1—C7—O2	−8.98 (17)	C31—C23—C24—C25	−179.28 (11)
C2—C1—C7—O2	170.78 (11)	C21—N1—C25—C24	−0.6 (2)
C6—C1—C7—O1	171.02 (10)	C23—C24—C25—N1	−0.4 (2)
C2—C1—C7—O1	−9.22 (15)	C30—N2—C26—C27	−0.28 (18)
C4—O3—C8—C9	179.75 (10)	N2—C26—C27—C28	−0.30 (19)
O3—C8—C9—C10	−176.05 (10)	C26—C27—C28—C29	1.34 (17)
C16—C11—C12—C13	0.19 (17)	C26—C27—C28—C32	−177.13 (10)
C17—C11—C12—C13	179.68 (10)	C27—C28—C29—C30	−1.82 (17)
C11—C12—C13—C14	0.47 (17)	C32—C28—C29—C30	176.57 (10)
C18—O6—C14—C15	5.71 (16)	C26—N2—C30—C29	−0.25 (17)
C18—O6—C14—C13	−175.08 (10)	C28—C29—C30—N2	1.35 (18)
C12—C13—C14—O6	−179.62 (10)	C24—C23—C31—C32	3.71 (17)
C12—C13—C14—C15	−0.37 (17)	C22—C23—C31—C32	−176.64 (10)
O6—C14—C15—C16	178.80 (10)	C29—C28—C32—C31	11.42 (17)
C13—C14—C15—C16	−0.39 (17)	C27—C28—C32—C31	−170.22 (10)
C14—C15—C16—C11	1.07 (18)	C23—C31—C32—C28	−179.72 (9)
C12—C11—C16—C15	−0.96 (17)		

### (II) 4-n-Propoxybenzoic acid–1,2-bis(pyridin-4-yl)ethane (2/1) Hydrogen-bond geometry (Å, º)

**Table:**

*D*—H···*A*	*D*—H	H···*A*	*D*···*A*	*D*—H···*A*
O1—H1···N1	0.966 (18)	1.657 (19)	2.6207 (17)	175.4 (16)
O4—H4···N2	1.010 (19)	1.610 (19)	2.6198 (17)	179 (2)
C6—H6···O2^i^	0.93	2.55	3.376 (2)	149
C27—H27···O5^ii^	0.93	2.52	3.389 (2)	156

Symmetry codes: (i) −*x*−2, −*y*, −*z*+1; (ii) −*x*+1, −*y*+1, −*z*+1.

### (III) 4-n-Butoxybenzoic acid–1,2-bis(pyridin-4-yl)ethane (2/1) Crystal data

**Table:**

2C_11_H_14_O_3_·C_12_H_12_N_2_	*Z* = 2
*M**_r_* = 572.68	*F*(000) = 612.00
Triclinic, *P*1	*D*_x_ = 1.289 Mg m^−^^3^
*a* = 7.702 (2) Å	Mo *K*α radiation, λ = 0.71075 Å
*b* = 10.726 (4) Å	Cell parameters from 15221 reflections
*c* = 19.010 (7) Å	θ = 3.0–30.2°
α = 83.861 (17)°	µ = 0.09 mm^−^^1^
β = 78.794 (16)°	*T* = 93 K
γ = 73.612 (15)°	Block, colorless
*V* = 1475.5 (9) Å^3^	0.40 × 0.20 × 0.10 mm

### (III) 4-n-Butoxybenzoic acid–1,2-bis(pyridin-4-yl)ethane (2/1) Data collection

**Table:**

Rigaku R-AXIS RAPIDII diffractometer	5219 reflections with *I* > 2σ(*I*)
Detector resolution: 10.000 pixels mm^-1^	*R*_int_ = 0.021
ω scans	θ_max_ = 27.5°, θ_min_ = 3.0°
Absorption correction: multi-scan (ABSCOR; Higashi, 1995)	*h* = −9→10
*T*_min_ = 0.768, *T*_max_ = 0.991	*k* = −12→13
14444 measured reflections	*l* = −24→24
6670 independent reflections	

### (III) 4-n-Butoxybenzoic acid–1,2-bis(pyridin-4-yl)ethane (2/1) Refinement

**Table:**

Refinement on *F*^2^	Primary atom site location: structure-invariant direct methods
Least-squares matrix: full	Secondary atom site location: difference Fourier map
*R*[*F*^2^ > 2σ(*F*^2^)] = 0.043	Hydrogen site location: mixed
*wR*(*F*^2^) = 0.129	H atoms treated by a mixture of independent and constrained refinement
*S* = 1.06	*w* = 1/[σ^2^(*F*_o_^2^) + (0.0705*P*)^2^ + 0.390*P*] where *P* = (*F*_o_^2^ + 2*F*_c_^2^)/3
6670 reflections	(Δ/σ)_max_ = 0.001
389 parameters	Δρ_max_ = 0.22 e Å^−^^3^
0 restraints	Δρ_min_ = −0.36 e Å^−^^3^

### (III) 4-n-Butoxybenzoic acid–1,2-bis(pyridin-4-yl)ethane (2/1) Special details

**Table:**

Geometry. All e.s.d.'s (except the e.s.d. in the dihedral angle between two l.s. planes) are estimated using the full covariance matrix. The cell e.s.d.'s are taken into account individually in the estimation of e.s.d.'s in distances, angles and torsion angles; correlations between e.s.d.'s in cell parameters are only used when they are defined by crystal symmetry. An approximate (isotropic) treatment of cell e.s.d.'s is used for estimating e.s.d.'s involving l.s. planes.
Refinement. Reflections were merged by *SHELXL* according to the crystal class for the calculation of statistics and refinement.

### (III) 4-n-Butoxybenzoic acid–1,2-bis(pyridin-4-yl)ethane (2/1) Fractional atomic coordinates and isotropic or equivalent isotropic displacement parameters (Å^2^)

**Table:**

	*x*	*y*	*z*	*U*_iso_*/*U*_eq_	
O1	0.51653 (13)	0.27771 (9)	0.61192 (5)	0.0218 (2)	
O2	0.64354 (14)	0.06698 (9)	0.59129 (5)	0.0236 (2)	
O3	0.68036 (13)	0.09242 (9)	0.92200 (5)	0.0209 (2)	
O4	0.22564 (13)	0.47777 (10)	−0.13269 (5)	0.0230 (2)	
O5	0.11681 (15)	0.69204 (10)	−0.11701 (5)	0.0309 (3)	
O6	0.08262 (14)	0.65267 (9)	−0.44591 (5)	0.0225 (2)	
N1	0.45623 (15)	0.30647 (11)	0.47936 (6)	0.0192 (2)	
N2	0.28494 (16)	0.44868 (12)	−0.00061 (6)	0.0218 (3)	
C1	0.62039 (17)	0.13761 (13)	0.70801 (7)	0.0163 (3)	
C2	0.56165 (17)	0.24210 (13)	0.75274 (7)	0.0182 (3)	
H2	0.5050	0.3265	0.7343	0.022*	
C3	0.58528 (18)	0.22352 (13)	0.82341 (7)	0.0193 (3)	
H3	0.5460	0.2953	0.8533	0.023*	
C4	0.66680 (17)	0.09943 (13)	0.85130 (7)	0.0178 (3)	
C5	0.72677 (18)	−0.00590 (13)	0.80742 (7)	0.0182 (3)	
H5	0.7830	−0.0904	0.8260	0.022*	
C6	0.70292 (17)	0.01481 (13)	0.73615 (7)	0.0176 (3)	
H6	0.7439	−0.0565	0.7059	0.021*	
C7	0.59569 (17)	0.15588 (13)	0.63141 (7)	0.0171 (3)	
C8	0.76407 (18)	−0.03082 (13)	0.95543 (7)	0.0191 (3)	
H8A	0.8937	−0.0635	0.9321	0.023*	
H8B	0.6977	−0.0957	0.9514	0.023*	
C9	0.75313 (18)	−0.00767 (14)	1.03313 (7)	0.0201 (3)	
H9A	0.6224	0.0241	1.0554	0.024*	
H9B	0.8140	0.0612	1.0358	0.024*	
C10	0.84284 (19)	−0.12942 (14)	1.07582 (7)	0.0234 (3)	
H10A	0.7823	−0.1985	1.0733	0.028*	
H10B	0.9738	−0.1611	1.0539	0.028*	
C11	0.8294 (2)	−0.10349 (16)	1.15397 (7)	0.0275 (3)	
H11A	0.7000	−0.0701	1.1755	0.041*	
H11B	0.8958	−0.0390	1.1568	0.041*	
H11C	0.8836	−0.1846	1.1800	0.041*	
C12	0.13736 (17)	0.61449 (13)	−0.23165 (7)	0.0186 (3)	
C13	0.14716 (18)	0.50757 (13)	−0.26965 (7)	0.0200 (3)	
H13	0.1674	0.4232	−0.2462	0.024*	
C14	0.12777 (18)	0.52337 (13)	−0.34095 (7)	0.0206 (3)	
H14	0.1331	0.4502	−0.3662	0.025*	
C15	0.10025 (17)	0.64686 (13)	−0.37591 (7)	0.0182 (3)	
C16	0.09327 (18)	0.75389 (13)	−0.33906 (7)	0.0192 (3)	
H16	0.0764	0.8379	−0.3629	0.023*	
C17	0.11117 (17)	0.73674 (13)	−0.26720 (7)	0.0189 (3)	
H17	0.1054	0.8099	−0.2418	0.023*	
C18	0.15721 (18)	0.60009 (14)	−0.15474 (7)	0.0205 (3)	
C19	0.02570 (19)	0.77902 (13)	−0.48132 (7)	0.0196 (3)	
H19A	0.1233	0.8246	−0.4871	0.024*	
H19B	−0.0871	0.8324	−0.4528	0.024*	
C20	−0.01042 (18)	0.75828 (13)	−0.55364 (7)	0.0199 (3)	
H20A	−0.0972	0.7036	−0.5473	0.024*	
H20B	0.1059	0.7114	−0.5831	0.024*	
C21	−0.0906 (2)	0.88721 (14)	−0.59280 (7)	0.0244 (3)	
H21A	−0.2101	0.9317	−0.5644	0.029*	
H21B	−0.0070	0.9437	−0.5964	0.029*	
C22	−0.1187 (2)	0.86963 (16)	−0.66777 (8)	0.0279 (3)	
H22A	0.0000	0.8288	−0.6968	0.042*	
H22B	−0.1723	0.9547	−0.6903	0.042*	
H22C	−0.2017	0.8141	−0.6645	0.042*	
C23	0.48692 (18)	0.19855 (13)	0.44432 (7)	0.0200 (3)	
H23	0.5283	0.1166	0.4688	0.024*	
C24	0.46126 (18)	0.20069 (13)	0.37437 (7)	0.0197 (3)	
H24	0.4851	0.1214	0.3517	0.024*	
C25	0.40048 (17)	0.31899 (13)	0.33696 (7)	0.0176 (3)	
C26	0.3688 (2)	0.43079 (13)	0.37340 (7)	0.0224 (3)	
H26	0.3279	0.5140	0.3502	0.027*	
C27	0.3969 (2)	0.42053 (14)	0.44380 (7)	0.0227 (3)	
H27	0.3729	0.4982	0.4680	0.027*	
C28	0.18349 (19)	0.54309 (15)	0.04192 (7)	0.0249 (3)	
H28	0.0936	0.6116	0.0230	0.030*	
C29	0.20326 (19)	0.54573 (14)	0.11249 (7)	0.0224 (3)	
H29	0.1278	0.6151	0.1409	0.027*	
C30	0.33331 (17)	0.44704 (13)	0.14174 (7)	0.0176 (3)	
C31	0.43955 (19)	0.34872 (13)	0.09681 (7)	0.0213 (3)	
H31	0.5310	0.2792	0.1141	0.026*	
C32	0.41101 (19)	0.35308 (14)	0.02703 (7)	0.0221 (3)	
H32	0.4842	0.2851	−0.0028	0.027*	
C33	0.37029 (19)	0.32021 (13)	0.26098 (7)	0.0203 (3)	
H33A	0.2547	0.2962	0.2622	0.024*	
H33B	0.4717	0.2525	0.2355	0.024*	
C34	0.35958 (18)	0.44973 (12)	0.21788 (7)	0.0174 (3)	
H34A	0.2562	0.5173	0.2425	0.021*	
H34B	0.4741	0.4749	0.2172	0.021*	
H1	0.497 (3)	0.288 (2)	0.5597 (13)	0.060 (7)*	
H4	0.247 (3)	0.468 (2)	−0.0805 (14)	0.069 (7)*	

### (III) 4-n-Butoxybenzoic acid–1,2-bis(pyridin-4-yl)ethane (2/1) Atomic displacement parameters (Å^2^)

**Table:**

	*U*^11^	*U*^22^	*U*^33^	*U*^12^	*U*^13^	*U*^23^
O1	0.0304 (5)	0.0184 (5)	0.0149 (5)	−0.0016 (4)	−0.0083 (4)	0.0015 (4)
O2	0.0355 (6)	0.0196 (5)	0.0155 (5)	−0.0052 (4)	−0.0069 (4)	−0.0011 (4)
O3	0.0293 (5)	0.0204 (5)	0.0122 (5)	−0.0023 (4)	−0.0087 (4)	0.0002 (4)
O4	0.0268 (5)	0.0238 (5)	0.0158 (5)	−0.0011 (4)	−0.0076 (4)	0.0015 (4)
O5	0.0420 (6)	0.0285 (6)	0.0196 (5)	−0.0005 (5)	−0.0105 (4)	−0.0044 (4)
O6	0.0329 (5)	0.0204 (5)	0.0124 (5)	−0.0031 (4)	−0.0067 (4)	0.0009 (4)
N1	0.0218 (6)	0.0222 (6)	0.0131 (5)	−0.0046 (5)	−0.0048 (4)	0.0013 (4)
N2	0.0261 (6)	0.0261 (6)	0.0139 (5)	−0.0077 (5)	−0.0062 (4)	0.0020 (4)
C1	0.0165 (6)	0.0193 (6)	0.0134 (6)	−0.0050 (5)	−0.0039 (4)	0.0012 (5)
C2	0.0203 (6)	0.0167 (6)	0.0166 (6)	−0.0032 (5)	−0.0052 (5)	0.0022 (5)
C3	0.0231 (7)	0.0183 (6)	0.0160 (6)	−0.0037 (5)	−0.0044 (5)	−0.0023 (5)
C4	0.0174 (6)	0.0231 (7)	0.0131 (6)	−0.0053 (5)	−0.0044 (4)	0.0006 (5)
C5	0.0197 (6)	0.0169 (6)	0.0167 (6)	−0.0020 (5)	−0.0055 (5)	0.0017 (5)
C6	0.0185 (6)	0.0177 (6)	0.0157 (6)	−0.0030 (5)	−0.0028 (5)	−0.0026 (5)
C7	0.0181 (6)	0.0188 (6)	0.0151 (6)	−0.0063 (5)	−0.0036 (4)	0.0013 (5)
C8	0.0204 (6)	0.0201 (7)	0.0163 (6)	−0.0028 (5)	−0.0069 (5)	0.0015 (5)
C9	0.0196 (6)	0.0257 (7)	0.0142 (6)	−0.0044 (5)	−0.0046 (5)	0.0017 (5)
C10	0.0245 (7)	0.0277 (7)	0.0158 (7)	−0.0040 (6)	−0.0055 (5)	0.0036 (5)
C11	0.0292 (8)	0.0367 (8)	0.0166 (7)	−0.0087 (6)	−0.0079 (5)	0.0049 (6)
C12	0.0151 (6)	0.0233 (7)	0.0152 (6)	−0.0017 (5)	−0.0037 (5)	0.0008 (5)
C13	0.0213 (7)	0.0196 (6)	0.0172 (6)	−0.0026 (5)	−0.0051 (5)	0.0025 (5)
C14	0.0253 (7)	0.0176 (6)	0.0184 (7)	−0.0036 (5)	−0.0053 (5)	−0.0018 (5)
C15	0.0180 (6)	0.0216 (7)	0.0129 (6)	−0.0024 (5)	−0.0029 (4)	0.0003 (5)
C16	0.0196 (6)	0.0191 (6)	0.0170 (6)	−0.0031 (5)	−0.0034 (5)	0.0020 (5)
C17	0.0186 (6)	0.0195 (6)	0.0182 (6)	−0.0033 (5)	−0.0042 (5)	−0.0026 (5)
C18	0.0181 (6)	0.0263 (7)	0.0155 (6)	−0.0029 (5)	−0.0043 (5)	0.0007 (5)
C19	0.0244 (7)	0.0187 (6)	0.0143 (6)	−0.0036 (5)	−0.0050 (5)	0.0020 (5)
C20	0.0223 (7)	0.0227 (7)	0.0141 (6)	−0.0049 (5)	−0.0044 (5)	0.0004 (5)
C21	0.0274 (7)	0.0260 (7)	0.0171 (7)	−0.0017 (6)	−0.0069 (5)	0.0011 (5)
C22	0.0291 (8)	0.0355 (8)	0.0200 (7)	−0.0086 (6)	−0.0102 (6)	0.0053 (6)
C23	0.0227 (7)	0.0197 (6)	0.0171 (6)	−0.0044 (5)	−0.0058 (5)	0.0026 (5)
C24	0.0239 (7)	0.0182 (6)	0.0174 (6)	−0.0054 (5)	−0.0051 (5)	−0.0005 (5)
C25	0.0169 (6)	0.0220 (7)	0.0137 (6)	−0.0055 (5)	−0.0029 (4)	0.0009 (5)
C26	0.0312 (7)	0.0177 (6)	0.0167 (7)	−0.0020 (6)	−0.0086 (5)	0.0015 (5)
C27	0.0312 (7)	0.0196 (7)	0.0166 (7)	−0.0032 (6)	−0.0077 (5)	−0.0018 (5)
C28	0.0250 (7)	0.0280 (7)	0.0189 (7)	−0.0005 (6)	−0.0079 (5)	0.0010 (5)
C29	0.0234 (7)	0.0246 (7)	0.0162 (6)	−0.0011 (6)	−0.0037 (5)	−0.0017 (5)
C30	0.0195 (6)	0.0215 (6)	0.0133 (6)	−0.0078 (5)	−0.0038 (5)	0.0010 (5)
C31	0.0246 (7)	0.0208 (7)	0.0170 (7)	−0.0020 (5)	−0.0069 (5)	0.0007 (5)
C32	0.0282 (7)	0.0224 (7)	0.0153 (6)	−0.0056 (6)	−0.0040 (5)	−0.0015 (5)
C33	0.0286 (7)	0.0202 (7)	0.0136 (6)	−0.0074 (5)	−0.0070 (5)	0.0004 (5)
C34	0.0220 (6)	0.0186 (6)	0.0123 (6)	−0.0049 (5)	−0.0051 (5)	−0.0010 (5)

### (III) 4-n-Butoxybenzoic acid–1,2-bis(pyridin-4-yl)ethane (2/1) Geometric parameters (Å, º)

**Table:**

O1—C7	1.3234 (16)	C14—C15	1.3962 (19)
O1—H1	1.02 (2)	C14—H14	0.9500
O2—C7	1.2144 (17)	C15—C16	1.391 (2)
O3—C4	1.3606 (16)	C16—C17	1.3871 (19)
O3—C8	1.4372 (16)	C16—H16	0.9500
O4—C18	1.3240 (17)	C17—H17	0.9500
O4—H4	1.03 (3)	C19—C20	1.5053 (18)
O5—C18	1.2146 (18)	C19—H19A	0.9900
O6—C15	1.3568 (16)	C19—H19B	0.9900
O6—C19	1.4374 (16)	C20—C21	1.5246 (19)
N1—C23	1.3374 (18)	C20—H20A	0.9900
N1—C27	1.3380 (18)	C20—H20B	0.9900
N2—C28	1.3314 (18)	C21—C22	1.521 (2)
N2—C32	1.3384 (18)	C21—H21A	0.9900
C1—C6	1.3906 (18)	C21—H21B	0.9900
C1—C2	1.3971 (18)	C22—H22A	0.9800
C1—C7	1.4903 (18)	C22—H22B	0.9800
C2—C3	1.3764 (18)	C22—H22C	0.9800
C2—H2	0.9500	C23—C24	1.3788 (19)
C3—C4	1.3977 (18)	C23—H23	0.9500
C3—H3	0.9500	C24—C25	1.3915 (18)
C4—C5	1.3939 (19)	C24—H24	0.9500
C5—C6	1.3887 (18)	C25—C26	1.3871 (19)
C5—H5	0.9500	C25—C33	1.5051 (18)
C6—H6	0.9500	C26—C27	1.3849 (19)
C8—C9	1.5062 (19)	C26—H26	0.9500
C8—H8A	0.9900	C27—H27	0.9500
C8—H8B	0.9900	C28—C29	1.3833 (19)
C9—C10	1.5243 (19)	C28—H28	0.9500
C9—H9A	0.9900	C29—C30	1.3875 (19)
C9—H9B	0.9900	C29—H29	0.9500
C10—C11	1.519 (2)	C30—C31	1.3946 (19)
C10—H10A	0.9900	C30—C34	1.5044 (18)
C10—H10B	0.9900	C31—C32	1.3799 (19)
C11—H11A	0.9800	C31—H31	0.9500
C11—H11B	0.9800	C32—H32	0.9500
C11—H11C	0.9800	C33—C34	1.5251 (19)
C12—C17	1.3895 (19)	C33—H33A	0.9900
C12—C13	1.396 (2)	C33—H33B	0.9900
C12—C18	1.4865 (18)	C34—H34A	0.9900
C13—C14	1.3796 (19)	C34—H34B	0.9900
C13—H13	0.9500		
			
C7—O1—H1	112.6 (13)	O5—C18—O4	124.19 (13)
C4—O3—C8	119.21 (10)	O5—C18—C12	122.87 (13)
C18—O4—H4	113.1 (13)	O4—C18—C12	112.93 (12)
C15—O6—C19	117.94 (10)	O6—C19—C20	107.13 (11)
C23—N1—C27	117.21 (12)	O6—C19—H19A	110.3
C28—N2—C32	117.67 (12)	C20—C19—H19A	110.3
C6—C1—C2	118.85 (12)	O6—C19—H19B	110.3
C6—C1—C7	119.97 (12)	C20—C19—H19B	110.3
C2—C1—C7	121.18 (12)	H19A—C19—H19B	108.5
C3—C2—C1	120.44 (12)	C19—C20—C21	111.41 (11)
C3—C2—H2	119.8	C19—C20—H20A	109.3
C1—C2—H2	119.8	C21—C20—H20A	109.3
C2—C3—C4	120.22 (12)	C19—C20—H20B	109.3
C2—C3—H3	119.9	C21—C20—H20B	109.3
C4—C3—H3	119.9	H20A—C20—H20B	108.0
O3—C4—C5	124.82 (12)	C22—C21—C20	112.54 (12)
O3—C4—C3	115.04 (11)	C22—C21—H21A	109.1
C5—C4—C3	120.13 (12)	C20—C21—H21A	109.1
C6—C5—C4	118.85 (12)	C22—C21—H21B	109.1
C6—C5—H5	120.6	C20—C21—H21B	109.1
C4—C5—H5	120.6	H21A—C21—H21B	107.8
C5—C6—C1	121.50 (12)	C21—C22—H22A	109.5
C5—C6—H6	119.3	C21—C22—H22B	109.5
C1—C6—H6	119.3	H22A—C22—H22B	109.5
O2—C7—O1	123.76 (12)	C21—C22—H22C	109.5
O2—C7—C1	122.85 (12)	H22A—C22—H22C	109.5
O1—C7—C1	113.38 (11)	H22B—C22—H22C	109.5
O3—C8—C9	106.52 (11)	N1—C23—C24	123.07 (12)
O3—C8—H8A	110.4	N1—C23—H23	118.5
C9—C8—H8A	110.4	C24—C23—H23	118.5
O3—C8—H8B	110.4	C23—C24—C25	120.01 (12)
C9—C8—H8B	110.4	C23—C24—H24	120.0
H8A—C8—H8B	108.6	C25—C24—H24	120.0
C8—C9—C10	112.97 (12)	C26—C25—C24	116.82 (12)
C8—C9—H9A	109.0	C26—C25—C33	123.58 (12)
C10—C9—H9A	109.0	C24—C25—C33	119.59 (12)
C8—C9—H9B	109.0	C27—C26—C25	119.72 (13)
C10—C9—H9B	109.0	C27—C26—H26	120.1
H9A—C9—H9B	107.8	C25—C26—H26	120.1
C11—C10—C9	111.88 (12)	N1—C27—C26	123.16 (13)
C11—C10—H10A	109.2	N1—C27—H27	118.4
C9—C10—H10A	109.2	C26—C27—H27	118.4
C11—C10—H10B	109.2	N2—C28—C29	122.87 (13)
C9—C10—H10B	109.2	N2—C28—H28	118.6
H10A—C10—H10B	107.9	C29—C28—H28	118.6
C10—C11—H11A	109.5	C28—C29—C30	119.95 (13)
C10—C11—H11B	109.5	C28—C29—H29	120.0
H11A—C11—H11B	109.5	C30—C29—H29	120.0
C10—C11—H11C	109.5	C29—C30—C31	116.87 (12)
H11A—C11—H11C	109.5	C29—C30—C34	120.72 (12)
H11B—C11—H11C	109.5	C31—C30—C34	122.39 (12)
C17—C12—C13	118.89 (12)	C32—C31—C30	119.61 (12)
C17—C12—C18	119.51 (12)	C32—C31—H31	120.2
C13—C12—C18	121.60 (12)	C30—C31—H31	120.2
C14—C13—C12	120.56 (13)	N2—C32—C31	123.03 (13)
C14—C13—H13	119.7	N2—C32—H32	118.5
C12—C13—H13	119.7	C31—C32—H32	118.5
C13—C14—C15	119.97 (13)	C25—C33—C34	115.09 (11)
C13—C14—H14	120.0	C25—C33—H33A	108.5
C15—C14—H14	120.0	C34—C33—H33A	108.5
O6—C15—C16	124.24 (12)	C25—C33—H33B	108.5
O6—C15—C14	115.68 (12)	C34—C33—H33B	108.5
C16—C15—C14	120.08 (12)	H33A—C33—H33B	107.5
C17—C16—C15	119.31 (12)	C30—C34—C33	113.82 (11)
C17—C16—H16	120.3	C30—C34—H34A	108.8
C15—C16—H16	120.3	C33—C34—H34A	108.8
C16—C17—C12	121.17 (12)	C30—C34—H34B	108.8
C16—C17—H17	119.4	C33—C34—H34B	108.8
C12—C17—H17	119.4	H34A—C34—H34B	107.7
			
C6—C1—C2—C3	0.01 (19)	C18—C12—C17—C16	179.58 (11)
C7—C1—C2—C3	179.91 (12)	C17—C12—C18—O5	14.7 (2)
C1—C2—C3—C4	−0.6 (2)	C13—C12—C18—O5	−166.26 (14)
C8—O3—C4—C5	0.84 (19)	C17—C12—C18—O4	−164.26 (12)
C8—O3—C4—C3	−179.33 (11)	C13—C12—C18—O4	14.75 (18)
C2—C3—C4—O3	−179.01 (12)	C15—O6—C19—C20	−171.63 (10)
C2—C3—C4—C5	0.8 (2)	O6—C19—C20—C21	174.20 (11)
O3—C4—C5—C6	179.42 (12)	C19—C20—C21—C22	176.83 (12)
C3—C4—C5—C6	−0.40 (19)	C27—N1—C23—C24	0.4 (2)
C4—C5—C6—C1	−0.23 (19)	N1—C23—C24—C25	−0.1 (2)
C2—C1—C6—C5	0.43 (19)	C23—C24—C25—C26	0.1 (2)
C7—C1—C6—C5	−179.47 (12)	C23—C24—C25—C33	−179.12 (12)
C6—C1—C7—O2	0.6 (2)	C24—C25—C26—C27	−0.4 (2)
C2—C1—C7—O2	−179.28 (13)	C33—C25—C26—C27	178.76 (13)
C6—C1—C7—O1	−179.56 (11)	C23—N1—C27—C26	−0.7 (2)
C2—C1—C7—O1	0.54 (17)	C25—C26—C27—N1	0.8 (2)
C4—O3—C8—C9	−178.27 (11)	C32—N2—C28—C29	−0.1 (2)
O3—C8—C9—C10	−178.06 (11)	N2—C28—C29—C30	0.0 (2)
C8—C9—C10—C11	−179.82 (12)	C28—C29—C30—C31	0.2 (2)
C17—C12—C13—C14	−1.19 (19)	C28—C29—C30—C34	178.69 (13)
C18—C12—C13—C14	179.80 (12)	C29—C30—C31—C32	−0.4 (2)
C12—C13—C14—C15	0.8 (2)	C34—C30—C31—C32	−178.80 (13)
C19—O6—C15—C16	−9.84 (19)	C28—N2—C32—C31	−0.1 (2)
C19—O6—C15—C14	170.46 (11)	C30—C31—C32—N2	0.3 (2)
C13—C14—C15—O6	−179.97 (11)	C26—C25—C33—C34	18.14 (19)
C13—C14—C15—C16	0.3 (2)	C24—C25—C33—C34	−162.70 (12)
O6—C15—C16—C17	179.36 (12)	C29—C30—C34—C33	133.61 (14)
C14—C15—C16—C17	−0.95 (19)	C31—C30—C34—C33	−48.02 (17)
C15—C16—C17—C12	0.52 (19)	C25—C33—C34—C30	178.79 (10)
C13—C12—C17—C16	0.54 (19)		

### (III) 4-n-Butoxybenzoic acid–1,2-bis(pyridin-4-yl)ethane (2/1) Hydrogen-bond geometry (Å, º)

*Cg*1 and *Cg*2 are the centroids of the C1–C6 and C12–C17 rings, respectively.

**Table:**

*D*—H···*A*	*D*—H	H···*A*	*D*···*A*	*D*—H···*A*
O1—H1···N1	1.02 (2)	1.60 (2)	2.6209 (18)	177 (2)
O4—H4···N2	1.03 (3)	1.58 (3)	2.6092 (18)	178.1 (19)
C32—H32···O3^i^	0.95	2.57	3.524 (2)	177
C11—H11*A*···*Cg*1^ii^	0.98	2.80	3.662 (2)	148
C33—H33*A*···*Cg*2^iii^	0.99	2.74	3.598 (2)	145

Symmetry codes: (i) *x*, *y*, *z*−1; (ii) −*x*+1, −*y*, −*z*+2; (iii) −*x*, −*y*+1, −*z*.
